# Addressing Substance Use and Misuse in East Texas: Stakeholder-Driven Needs and Priorities

**DOI:** 10.3390/ijerph192215215

**Published:** 2022-11-18

**Authors:** Yordanos M. Tiruneh, Kimberly S. Elliott, Linda Oyer, Emmanuel Elueze, Vanessa Casanova

**Affiliations:** 1 Department of Preventive Medicine and Population Health, School of Medicine, University of Texas at Tyler, Tyler, TX 75708, USA; 2 Department of Internal Medicine, Division of Infectious Diseases, University of Texas Southwestern Medical Center, Dallas, TX 75390, USA; 3 Department of Health Policy, Economics, and Management, School of Health Professions, University of Texas at Tyler, Tyler, TX 75708, USA; 4 East Texas Council on Alcoholism and Drug Abuse, Longview, TX 75601, USA; 5 Graduate Medical Education and Designated Institutional Official, School of Medicine, The University of Texas at Tyler, Tyler, TX 75708, USA; 6 Department of Occupational and Environmental Medicine, School of Medicine, University of Texas at Tyler, Tyler, TX 75708, USA

**Keywords:** substance use, substance misuse, SUD, treatment, prevention, recovery, community based, community health, public health, stakeholders

## Abstract

Background—This project sought to assess needs, perceived challenges, and priorities regarding substance use disorder (SUD) in East Texas and develop a community-driven research agenda to address those challenges. Methods—Data were gathered through nine focus-group discussions (FGDs) with stakeholders: people living with SUD, families, medical providers, counselors, representatives of community-based organizations, and law enforcement officers. We asked participants how substance use manifests in their communities, which challenges they confronted in coping with substance use and misuse, and in which order their needs should be prioritized. Findings were reported at community forums to confirm the list of challenges and prioritize needs. Results—Five themes emerged from the FGDs indicating major challenges: (a) access to SUD treatment and recovery resources, (b) mental health and resiliency, (c) education, training, and professional development to facilitate treatments, (d) care and service coordination, and (e) community/social support for people living with SUD and their families. Conclusions—Significant resources such as financing, collaboration across silos, and community education are needed to effectively manage this public health problem. Our findings can inform research and outreach to help East Texans develop interventions, research programs, and educational opportunities for clinicians, community-based organizations, law enforcement officers, and counselors to build capacity for SUD prevention, treatment, and recovery.

## 1. Introduction

Rising levels of substance abuse have been spreading throughout the United States for more than a decade, often in areas that had in the past avoided the worst outcomes of substance use disorder (SUD) [1]. In October 2017, the U.S. Department of Health and Human Services officially declared a public health emergency to address the “opioid crisis” [2]. In 2020, overdose deaths related to any opioid, including prescription opioids, heroin, and fentanyl, claimed the lives of almost 69,000 people in the United States, accounting for the majority of drug-related deaths [3,4]. This epidemic has affected both urban and rural communities with equal intensity. While overdose rates remain higher in urban areas, the rates are increasing at a higher rate in rural areas [5]. In 2020, eight states suffered higher drug overdose rates in rural counties as compared with urban counties [3].

Differences in population densities between urban and rural communities, however, make it reasonable to infer that the epidemic has taken a heavier toll on rural than urban communities. While all regions face significant challenges, those faced by rural communities include unique access-to-care issues. Lack of access to care is only one of many barriers that make preventing and treating SUD in rural communities difficult. Social issues, such as stigma and fear of criminalization, have grown in salience while systemic issues such as underinsurance or lack of insurance, lack of treatment facilities, lack of trained health professionals, and poor coordination of care complicate mitigation efforts [6].

Texas is among the worst states with respect to illicit drug use among people aged 18 years and older. The state also ranks near the top in marijuana use among persons aged 12 years and older [7]. East Texas exhibits a higher rate of substance use than the rest of the state. According to the Regional Needs Assessment conducted by the Prevention Resource Center, the total rate of drug-related arrests for adults from 2018 through 2020 in Public Health Region 4 of Texas (701.2 per 100 k) was higher than that for the entire state (634.2 per 100 k) [8]. Also, compared with the rest of the state, slightly higher percentages of East Texas students (grades 7–12) reported their first use, before reaching adulthood, of tobacco (12.9% East Texas:12.8% Texas), marijuana (12.9% East Texas:12.8% Texas), cocaine (14.8% East Texas:14.3% Texas), ecstasy (15.2% East Texas:14.6% Texas), methamphetamine (14.1% East Texas:13.6% Texas) and synthetic marijuana (13.9% East Texas:13.8% Texas) [8].

To curb the growing use of such substances, we must first understand the cycle of use and misuse. These behaviors are often associated with social exposure to smoking or alcohol. Among youth and adolescents, correlations have been found between the initial use of marijuana and subsequent alcohol and illicit drug use [9]. This theory that marijuana and alcohol serve as gateways to the use of more difficult-to-control drugs has produced associations in the literature that vary in strength [10]. The cycle of substance use has also been linked to exposure to smoking and drinking within both social groups and families [10]. A study has associated exposure to peers or parents who drink or smoke with a high likelihood of eliciting similar behaviors among 12–17-year-old youth [11]. It is therefore important to understand the social context in which young people engage in substance use if we are to create effective prevention strategies [10].

Combatting the cycle of abuse faces a significant challenge. East Texas lacks long-term treatment facilities [8]. Decision-makers in the region must make strategic decisions with limited resources despite significant gaps in data. Current data gaps involve underrepresented populations (the homeless, the institutionalized, the LGBTQ community, non-English-speaking persons, and undocumented residents), hospital discharges for youth substance overdose/poisoning, and ER admissions of adolescents experiencing health crises associated with alcohol, tobacco, opioids, or other drugs as well as behavioral health crises [8].

Studies have shown that the most effective interventions have involved community stakeholder collaboration and input. SUD is a public health issue and addressing this epidemic requires multiple stakeholders working in conjunction with public health institutions. Studies have also shown that the most effective opioid-cessation programs have indeed included efforts contributed by a broad range of community stakeholders. To address barriers raised by the stigma associated with SUD as well as fear of criminalization, cities like Burlington, Vermont and Gloucester, Massachusetts have designed programs that actively involve law enforcement officers in key roles. The Gloucester program provided sites where users can turn in drugs and syringes anonymously and coordinated with clinical services to provide medications that treat opioid use disorder [12]. Similarly, a county-wide effort carried out in Burlington demonstrated that using police officers as public health or public safety agents using evidence-based approaches led to significant reductions (50%) in fatal overdose deaths [13]. The Gloucester program was found to be effective in connecting participants with needed care, but it was not as effective in sustaining consistent preventive treatment [12]. 

The challenges to SUD treatment and prevention reflect inefficiencies in a fragmented healthcare system. It is important to broaden the range of stakeholders who should be involved in addressing this issue and engage community members beyond healthcare providers and public health officials. Law enforcement agencies, educational institutions, and communities in general must be involved in conversations addressing the challenges of and suggesting solutions to substance misuse including the opioid epidemics plaguing both urban and rural communities [14].

The goal of this project was to engage community stakeholders and explore needs, perceived challenges, and priorities regarding substance use in East Texas and to develop a community-driven research agenda to address the associated problems. We asked community stakeholders to share their opinions regarding (1) how substance use is manifested in East Texas, (2) which concerns or challenges they confront in coping with SUD, and (3) which priorities their communities should adopt to address these needs.

## 2. Materials and Methods

We engaged with stakeholders using a community-based participatory research (CBPR) framework to facilitate collaboration between community stakeholders and academic researchers [15]. Within the CBPR framework, we engaged with a diverse mix of relevant stakeholders, including people living with SUD and their family members, in the inception, conduct, and the analysis phases. Data were gathered through nine focus-group discussions (FGDs) using semi-structured interview questions with probes to assess the substantive content of verbally expressed opinions, attitudes, and experiences. [16]. Three main questions informed the data-gathering process. First, we asked participants how SUD manifests in East Texas and whether it is a concerning problem in the broader community. Through this question we explored the extent and nature of the problem, enabling us to identify the most commonly used substances, the most severely affected demographic group/s, and where members of the community seek help. We then asked stakeholders to share the concerns and challenges they confront in addressing substance use in the region, focusing on their specific experiences with prevention, treatment, and/or recovery. Finally, based on a list of problems/challenges that framed the discussions, we asked participants to identify, in order of priority, those they found most concerning. The probing questions we used to prioritize needs included: (a) Which specific needs/problems should be prioritized? (b) Is there anything in particular you would like to see change? and (c) Which needs should be addressed immediately?

### 2.1. Participants

Focus groups were constructed to represent diverse populations with SUD-related experience, including two focus groups with participants who live with or had lived experience with SUD, one with families/caregivers, one with community-based organizations working on SUD, one with individual counselors and social workers, one with clinicians/medical providers, and one with law enforcement officers.

Through convenience sampling, individuals living with SUDs and families were recruited from community-based organizations engaged in programs that provide services and support. All of the FGDs were conducted in English and were held virtually. To be included in the group consisting of those living with SUD and their families, participants needed to have access to an internet-enabled device or phone and had to be able to speak English. Participants in the other FGDs were recruited by the Steering Committee members of the Community-Academic Partnership for Substance Abuse in East Texas (CAPSA-ET), a community-academic consortium for substance use and misuse. All the FGDs were audiotaped with participants’ verbal consent. All FGDs were facilitated by three or four members of CAPSA (one or two academic partners, one community partner, and a note taker). Human-subject institutional review board approval was obtained from the University of Texas Health Science Center at Tyler (HSC-21-009). Table 1 lists the pertinent demographics of the participating groups.

### 2.2. Focus Group Procedure

Nine FGDs were conducted virtually through an online platform. A semi-structured focus group guide was used to generate conversations about substance use in East Texas and to identify concerns/challenges with which participants were familiar when addressing SUDs in the region. These discussions lasted about 45 min each. In the final 15–30 min of each discussion, a list of responses to the concerns/challenges were presented by facilitators, and discussants were asked to prioritize and provide rationales for their rankings. The three faculty members comprising the research team facilitated discussions in turn, with a community partner and a qualitative researcher who took notes. Before each focus group discussion began, a moderator led introductions and confirmed with the group that the discussion would be audio-taped and that those whose responses were included in the transcripts would be de-identified prior to sharing the data with the study team for analysis.

### 2.3. Content Analysis

The de-identified transcripts were analyzed to identify emergent themes through two stages of content analysis using matrix techniques [17]:

(1) Coding by one researcher to create an initial coding structure grounded in the discussion topics described above, and
(2) A team process carried out to verify and expand the coding matrix to include sorting of discussions into emergent themes and categories.

The final coding structure was then shared with all other members of the research team, who served as external auditors. Our final consensus regarding the results was presented at a community forum in a peer debriefing.

### 2.4. Member Checking

The research team validated the preliminary findings using formal member-checking procedures [18,19,20]. We conducted four community (formal member-check) forums, with each including as many as 13 participants. These forums validated the preliminary findings and helped us determine the priority order of the needs. Each forum lasted approximately one-and-a-half hours. The first half of each session included a PowerPoint presentation of the project, with a concentration on preliminary findings, especially the challenges list generated in the content analysis. The remainder of the time was devoted to eliciting feedback, as we asked attendees, “Have we missed anything?” and “How do we prioritize our needs?” Attendees at the community forums were identified and invited by CAPSA Steering Committee members. The four groups comprised (1) people in recovery or who had lived experience with SUDs, patient advocates, and families; (2) a mix of community-based organizations (CBOs), counselors, and representatives of law enforcement; (3) members of regional behavioral health and SUD teams/coalitions; and (4) CAPSA-ET steering committee and Advisory Council members.

## 3. Results

The various stakeholder groups expressed a wide range of concerns regarding substance use. Those in recovery discussed individual challenges—lack of resiliency and coping strategies, the ready availability of drugs as a means of entertainment and stimulus, and the pathology of addiction—more extensively than other groups. The law enforcement focus group concentrated largely on criminal implications while those with counselors and providers were heavily patient-focused. The FGD with CBOs was mostly community focused. Topics that were common to all three areas of focus—individual experiences, patient treatment, and criminality—were few and narrow. Commonalities included (1) the overwhelming volume of drugs in communities; (2) inadequate provision of prevention, treatment, and recovery services or services being at maximum capacity; (3) a general lack of awareness of available resources and programs; and (4) lack of training enabling practitioners and law enforcement personnel to address the problem effectively. The CBO and law enforcement FGDs revealed more common concerns than did those involving counselors or providers. CBOs and counselors/providers seemed to share concerns most extensively. Families and individuals concerned with SUD closely mirrored counselors/providers and then CBOs, especially regarding resource needs.

There were also clear and distinct differences between the groups rooted in their respective perceptions of the SUD problem through their professional lenses. Even in mixed-group discussions, a shared vision was neither expressed nor sought. In particular, when asked to prioritize challenges, participants in each group continued to focus on their own professional concerns while not acknowledging those among other groups.

### 3.1. The SUD Context in East Texas

All of the FGD participants shared the view that SUD is a serious problem in East Texas that is fueled by easy and cheap access to drugs resulting from (1) its geographical location (as a main conduit for drugs destined for metropolitan Dallas) and, (2) the region’s culture of acceptance of alcohol use, smoking, and recreational use of drugs. Participants mentioned that drinking alcohol with parents, vaping, and using marijuana, which are mentioned as gateway behaviors that lead to abuse of harder substances, are often normalized in East Texas. In particular, the FGD with counselors revealed that families are permissive of such activities and consequently it can be difficult to promote prevention activities among East Texas youth: “And then also the enablers. You’ve got clients that their family members will come in and they’re giving it to them. Oh, they’re not that bad”.

Although most FGD groups were unable to quantify the problem with evidence, methamphetamines (meth), designer crack cocaine, and opioids were all identified as commonly used in the region. All groups agreed that the meth is the drug used most commonly. All FGD participants shared the view that the trend in drug use is changing rapidly with new and more dangerous drugs becoming available. Recent trends also indicate an increase in the availability of synthetic drugs. Additionally, most participants mentioned that they find it challenging to keep up with the rapidly evolving mix of drugs and changing trends in illicit drug use: “I used to feel like East Texas, we were behind a little bit and that the Dallas area or Houston area, they would get something first and then we had a little time and I’d know about it. Things are coming so fast here that it’s happening there and here…” Some participants also shared that relatively new forms of substance use such as vaping are particularly attractive to younger consumers as many find the fruit flavors appealing.

### 3.2. Major Challenges with SUD in East Texas

Five themes emerge from our FGDs as major concerns, challenges, resiliencies, and/or priorities related to SUD in East Texas. These include (a) access to SUD treatment and recovery resources, (b) mental health and resiliency, (c) education, training, and professional development to facilitate SUD treatments, (d) care and service coordination, and (e) community/social support for people living with SUD and their families. We summarize the five themes with illustrative quotes in Appendix A.

#### 3.2.1. Access to SUD Prevention, Treatment, and Recovery Resources

The theme of access to resources captures all the challenges mentioned related to SUD prevention, treatment, and recovery. First, all FGD participants noted the insufficiency of resources to support prevention of, treatment of, and recovery from SUD in East Texas, identifying availability of access as an important dimension. Second, accessing the region’s scant treatment and recovery resources is difficult for patients and families and even for providers seeking to make the necessary referrals, as affected individuals do not know which programs are available or where to find them. One care provider mentioned this difficulty: “Knowing where to send patients for detox would be helpful; places to get people medically stable.” In-patient treatment facilities offer very few beds and access is severely limited. Most treatment facilities are at maximum capacity for in-patient accommodations; patients often must wait months for open beds, delaying and or otherwise affecting recovery outcomes. Equally important are resources for transitional care following detox treatments, such as sober-living and long-term recovery facilities.

Recovery resources/programs are hard to find or barely known in East Texas communities, even for care providers. Moreover, there are too few such resources to meet community needs and they involve lengthy admission processes with rigorous criteria that must be met to qualify for care. Mental health counseling, spiritual counseling, and faith-based programs are similarly scarce. In particular, access to long-term recovery was mentioned by all groups as a formidable challenge. As one participant explained: “It’s frustrating, because from what my experience was, solely, with the exception of the sober living house that I have found, other than that and the 12-step programs, there was nothing to get me sober. I had no support from anybody, and I had to do this on my own and accept that I was gonna be alone on this until I could turn the tables around. And so, it’s frustrating because there’s supposed to be this movement to assist those with substance abuse disorder.” Similarly, another patient living with SUD stated, “it’s baffling to me just how easy it is to find XYX drugs. And the sad thing is about that is that, on the flip side for recovery, from what I’ve seen and what—I’ve asked others around me—it’s hard to find organizations or recovery institutions or something like that in this area to assist with recovery that’s outside of the sober living program”.

#### 3.2.2. Mental Health and Resiliency

Most drug use begins as a coping mechanism in response to a stressful life event or a lack of personal resources with which to address or respond to boredom for lack of employment or educational opportunities. Young people in particular find such conditions challenging as they know little about self-care and their health-seeking behavior is generally poor. Unattended mental health conditions often trigger substance use, especially among youth. As one counselor put it: “I also think it would be interesting if instead of just learning about the dangers they could learn about what are some alternative coping skills because I am always shocked and heartbroken when I do presentations to students and I ask them why do you think your peers are drinking. And I always get at least one student who says that they’re trying to shut off their feelings.” Mental illness was mentioned as a common cause of substance use as well as a major challenge for treating SUD in East Texas. An SUD is often a symptom of underlying mental health issues and unless effective mental healthcare facilities are in place in a given community, we are treating symptoms only. Most FGD groups highlighted the need to strengthen prevention efforts in the East Texas region as educational resources for families are inadequate. Educational outreach, especially aimed at youth, should be current and should employ attractive strategies to meet the needs of younger generations. Some participants also mentioned that addressing social determinants of health could be crucial for prevention as these factors are “perpetrators of SUD.” In East Texas, most young people who use substances live in low-socioeconomic-status neighborhoods and are at greater risk because of struggles and daily stressors that reflect their life circumstances.

#### 3.2.3. Education, Training, and Professional Development for SUD Treatments

FGD participants shared that, in their observations, most current SUD treatment programs do not work and there are no effective strategies for treating relapses. Youth recovery programs are not as well attended as some treatment/prevention strategies, such as DARE, are not effective. Most FGD groups identified a lack of current knowledge or up-to-date training as challenges to efforts to address SUD in East Texas. Education regarding existing resources, the dynamics of SUD, categories of addiction-related substances, and effective treatment strategies are greatly needed. An issue that cut across groups involves the need to increase the effectiveness of treatment strategies. This issue has been aggravated to an even greater extent by the COVID-19 pandemic. Participants shared that it is well known in the recovery field that isolation “is not good.” Unsurprisingly, forced isolation as a result of the pandemic has had a devasting effect on those living with SUD in East Texas, with “so many elements that choke people down.” The region needs additional training opportunities that would help providers and counselors consider the effects of the pandemic on the health behaviors of persons living with SUD.

The clinicians’ focus group also mentioned a lack of providers with expertise in SUD and emphasized the need for training in SUD diagnosis and treatment, medication-assisted therapy, and effective communication with individuals living with substance use: “I would say my challenge is I don’t have the skillset. […] It was just never part of my training. I mean we knew how to detox somebody in the ICU or on the floor. But dealing with somebody in a longitudinal way was just never part of our training and it seems like it’s not as accessible as it needs to be…” Likewise, law enforcement FGD participants highlighted the need for funding to train officers regarding drugs and to enable them to apply safety measures to assist them in managing intoxicated individuals: “And I think they’re well prepared but there are certainly some challenges in getting everybody trained in substance abuse and de-escalation and everything else that’s thrown at us so it’s a big challenge to get it all done…”

The counselors’ FGD also revealed a need for ongoing training and education regarding the evolving nature of drugs and new trends in drug use. “So, you can never say we’re fully trained and prepared for everything because as soon as you say that you get something thrown to you from left field. So, I mean our officers I feel like we are trying to get the best training we can. We’re open and we’d love to have as much training as we can to make sure we’re as prepared as we can be. It’s always a challenge…” The need for more education on relapses and effective treatments for those with dual diagnoses was mentioned, as mixing recreational substances with psychotropic medications can be deadly.

Some stakeholder groups, especially families giving care to individuals living with SUD, those who are counselors, and those representing CBOs, emphasized the need to expand education to help families cope with SUDs and stigma. This effort was related directly to prevention efforts, especially among youth. Education for families should focus on prevention as well as early intervention by teaching families the signs indicating that a child is suffering from SUD and guidance regarding how to obtain help and stay well.

The need for data with which to characterize the SUD problem in East Texas and to improve outcomes with evidence-based treatment options was discussed by the providers, CBOs, and counselors as well as the community forum participants. Participants highlighted the need for a surveillance system to parse out what is missing and what is already being captured in real time if possible, to compare treatment options, and to improve services to address SUDs in the region. One forum participant mentioned that “data enriches all the entities”—law enforcement, counselors, providers, and others. “Data put us on the same page, strengthen the community and tie us all together.” One forum participant suggested creating a dashboard to monitor the magnitude of the problem and reveal SUD patterns in East Texas to help mirror education and outreach efforts in the local community. Such data would be useful for improving awareness as well as obtaining funding for SUD programs.

#### 3.2.4. Care and Service Coordination

Many of the FGD participants mentioned being unaware of existing resources for treatment of and recovery from SUD. This information gap, especially regarding information related to access and affordability, makes navigating existing resources difficult. For example, the clinicians’ FGD revealed that providers are not aware of many existing resources in the community and thus could not help patients navigate the system beyond the clinic set-up. Law enforcement officers mentioned a similar concern, noting that the criminal justice system (CJS) has become a revolving door for people who engage in substance use. SUD drives many crimes as well as homelessness and family disruptions. The same population moves in and out of the CJS as there are no proper treatment or recovery resources during incarceration and even scarcer or non-existent for people reentering the community after incarceration. Law enforcement officers mentioned challenges they face navigating the healthcare system and linking people with community resources for treatment or recovery: “A lot of them don’t know where to turn to, where to get those resources. I don’t know. I don’t know if it’s an education thing with their probation officer if they don’t know where to get the resources from or parole officer. They don’t know where to go to…” A forum participant mentioned that we “need [a] better medical system put into place that will allow [people in need] to secure help quicker. All agencies are separate and require different paperwork.” This issue, which we term “coordination of care,” was mentioned as vital to avoiding duplication of effort in programming and finding social support and programs that can facilitate community re-integration more effectively. Several participants suggested that creating a comprehensive list of resources in the area, including treatment facilities and programs, transportation options, childcare facilities or services, affordable mental health services, and other social services, would benefit multiple stakeholders.

#### 3.2.5. Community/Social Support for Individuals Living with SUD and Families

Social support and community programs that help individuals living with SUD are identified as vital to ensuring continuity of care. Variety and availability were mentioned as key features of these resources. Discussants represented an array of community members and, unsurprisingly, endorsed an equally diverse array of community/social support programs. These included programs that address patient-driven needs for mental health counseling to learn positive coping strategies; family-focused educational programs to promote both prevention and recovery among youth; and faith-based and spiritual counseling programs pressed by many patient-advocate organizations. Specific mention was made of community reentry programs for people who are released from incarceration as well as for military veterans returning from service. Moreover, families as well as those living with SUD may have fewer social-support options outside of their local communities. One family member remarked, “There’s not very many support services for the families, to teach them what to do, to teach them not to enable and give them support and let them know they’re not out here by themselves…and so I think there needs to be more resources for families to go and get the support and education on what to do to help their addict or alcoholic person and their family. Or how to help themselves stay sane, you know?”.

Given the severe stigma that might be associated with substance use and misuse, traditional recovery and support programs have remained successful because they guarantee anonymity and therefore build trust through regular meetings. This is not necessarily possible in smaller communities. The lower population density of rural East Texas communities challenges maintaining anonymity when seeking recovery support services. Hence, people living with SUD as well as their families may not feel comfortable participating in any of the few existing support groups for fear of being recognized. There are also smaller communities where these services do not exist at all. For those living in these communities, physical access to recovery services, a logistical issue, reflects difficulties with travel and planning and therefore matters to a greater extent than anonymity.

Perhaps ironically, the COVID-19 pandemic has to some degree reduced the salience of anonymity, as persons living with SUD and their families have benefited from increased access to online recovery support meetings that often involve participants residing across a wide range of localities. Nevertheless, there is an intangible personal quality that participants experience during in-person meetings that is difficult if not impossible to recreate in the modality of virtual recovery and family support meetings. One participant in the counsellor FGD said: “There are some like we’re hearing right now because everything is virtual and kids are having to attend classes like for MIPs and stuff virtually that some of them are—the requirements have changed.” Thus, these options entail clear person-specific pros and cons that are based on individual preferences. Rural East Texas communities tend to be close-knit. Many social circles remain intact from childhood into adulthood, with few new acquaintances added. Therefore, the challenge for people in recovery who return from incarceration or from military service is that the recovery experience can be socially isolating, particularly if some in one’s social circle continue to consume drugs and alcohol. Recidivism is likely to occur in the absence of the support offered by recovery groups.

## 4. Discussion

This study sought input from a diverse range of stakeholders to identify community-driven needs and priorities related to substance use in East Texas guided by the CBPR framework. The study highlights three main findings. First, we identified several salient geographic and cultural features that contribute to easy, cheap access to substances in East Texas, highlighting the imperative to address those features in prevention and treatment efforts. Second, we found that the main challenges involved in addressing SUD in East Texas are limited access to resources for SUD treatment and recovery; lack of training/education on SUD prevention, treatment, and recovery for providers; lack of care and service coordination (navigation) among community stakeholders; the need for mental health and resiliency services; and lack of support for families and caregivers. Third, we observed that the needs and challenges identified by various stakeholder groups often reflect distinctive or unique features of particular stakeholder groups, thus indicating the need for comprehensive explorative studies to inform research and outreach undertaken to provide more effective care for individuals with SUD and their families in East Texas.

Our data identified a culture of tolerance for substance use in East Texas as a key challenge in the struggle to address SUD in the region. Some participants, particularly the counselors, emphasized that their efforts to prevent substance use is often hampered by parents who allow teenagers to try alcohol or smoking at an early age. While such a cultural environment promotes substance use, the stigma associated with substance use in rural communities—which experience stigma at a “more pronounced” level [5]—discourages persons living with SUD from seeking care. The anonymity offered when seeking treatment in urban areas is largely absent from rural communities. Studies have shown that there are in fact cultural differences between rural and urban populations that affect how SUD is treated. These issues could potentially not only cause those living with SUD in rural communities to avoid care but also subject them to a higher risk of overdose [21] or continued substance misuse, which might include exposure to infectious diseases such as HIV through high-risk injection behaviors [5,22].

Participants portrayed East Texas as a region comprising small rural communities where a scarcity of recreational opportunities facilitates substance use. In addition to its rurality, East Texas features two major interstate highways that connect three major cities. The route from Shreveport, Louisiana, to Dallas on I-20 and the route from Houston to Dallas on I-45 both traverse East Texas, affording East Texas communities easy access to illicit drugs and human trafficking. In a 20-state study of labor trafficking between 2013 and 2016, Texas (34.4%) was found to have the highest percentage of related arrests [23]. According to a 2003 National Drug Intelligence Center report [24], Texas is a national distribution center for illicit drugs. Interstate 20 provides a direct route through Dallas to Shreveport; Birmingham, Alabama; and Atlanta, Georgia [24]. Thus, East Texas communities lie squarely in the path over which illicit drugs are transported, making them readily available. Still, access to and use of illicit drugs is only part of the supply issue. Studies have shown in fact that prescription drugs cause the greatest share (31%) of drug overdose deaths in rural communities [5]. This ready supply of substances (illicit and prescription drugs) makes it all the more challenging for those living with SUD in East Texas to escape the cycle.

Our participants across stakeholder groups highlighted the lack of adequate care and service as the main challenge to combatting SUD in the region, especially for long-term treatment and recovery. Further exacerbating the lack of resources is the difficulty accessing or even knowing about existing resources. SUD programs in East Texas largely lack the full range of services that clients seeking treatment need, resulting in discontinuity of care. Even when a detox bed is found, for example, sober-living facilities and long-term rehabilitation services after a patient is released are scarce. While this is a challenge faced by many areas across the nation, it is particularly pervasive among rural communities as compared with their urban counterparts. Often, in sparsely populated rural areas, specialty healthcare service is not physically accessible to many of the rural residents [25,26].

Some population groups find themselves even more severely disadvantaged with respect to the care and support services they need. Our participants highlighted the unique challenges that people passing through the CJS who need treatment for SUD face, which they attributed to high relapse and recidivism rates. Along with housing and food insecurities, recently incarcerated persons living with SUD often face complications accessing follow-up healthcare coverage, and therefore treatment and prevention services are disrupted after their release, likely contributing to recidivism [27,28].

Our study participants provided insights into challenges to treatment that are often identified in the literature—such difficulty navigating the healthcare system. Englander et al. [25] reported that many hospitals neither treat SUD during hospitalization nor make referrals to treatment facilities. This failure to connect SUD patients to relevant treatment services may reflect a lack of resources such as training and financial support [29]. While more healthcare facilities see SUD treatment as an outpatient service, efforts are needed to incentivize hospitals to support patients by connecting them to outpatient treatment and recovery services. Some recommend that hospitalists receive training in addiction medicine as well as incentives to complete buprenorphine waiver training [29].

Our study findings indicate that addressing dual diagnoses of mental health and SUD is a major challenge in East Texas. Participants discussed the importance of empowering the community with mental health resiliencies, especially for the younger generation. Such empowerment would include mechanisms for coping with stress, access to mental health services, addressing mental health stigma, and access to community resources and social support structures. Empowering providers to offer care for dual-diagnosis patients is important to address co-occurring conditions. This finding is consistent with those reported in other studies in rural areas revealing gaps in care for dual psychological and SUD diagnoses that can lead to poor treatment outcomes as well as higher admission rates, delayed symptom remission, and increased risk of suicide [30,31]. An already under-resourced rural healthcare system cannot prevent such outcomes.

In line with previous research, our focus group participants who are involved in SUD prevention and treatment services indicate that a lack of up-to-date training hinders providers’ capacity to deliver services; therefore they stress the need for better and more comprehensive provider education. For instance, participants in our counselor focus group emphasized that the rapidly changing characteristics of drugs, especially those related to vaping, are difficult to track, and they struggle to stay up to date, particularly regarding their effects on adolescents.

Addressing the needs of persons suffering from SUD is complex and requires close collaboration among community stakeholders, especially when the resources needed to care for and support them are so scarce. Studies have revealed the need for efforts to foster collaboration and coordination among community stakeholders across functional healthcare, social service, and peer support service silos to address the “complex system needs” of those living with SUD [32]. Providers can benefit from understanding how experiences with stigmatizing behavior at key points of care might fuel the cycle of misuse because it can essentially weaken motivations to seek prevention, treatment, or recovery services. Understanding these potential barriers to treatment and recovery creates opportunities for training within the affected healthcare and social service organizations [33].

Our study of the perceived challenges related to SUD in East Texas should facilitate the prioritization of community mitigation efforts both in this and similar rural regions. The next step is to identify through further research which of these priorities is actionable. The identified concerns emerged as possible topics for developing effective interventions, research programs, and educational opportunities enabling clinicians, community-based organizations, law enforcement officers, and counselors to build their capacity to provide optimal care. These include (a) effective educational and counseling interventions for self-regulation and positive coping mechanisms; (b) community reentry programs after incarceration; (c) comparing the effectiveness of SUD treatments; (d) developing ways of engaging families in prevention, treatment, and recovery practices; and (e) training law enforcement officers in screening, managing, and finding follow-up care for individuals who use substances. More rigorous research is needed to assess barriers and identify facilitators in the effort to implement more streamlined, full-range care for SUD.

Improving healthcare delivery and community support for SUD effectively requires both community engagement and data-driven metrics. By adopting a data-driven approach, community and academic partnerships can triangulate with evidence-based approaches to address SUD needs equitably while respecting cultural differences [34]. Data-driven approaches to addressing SUD and up-to-date information on newer substances also contribute to effective SUD prevention and treatment. For instance, the data on the physiological and developmental effects of vaping are still relatively new and more data are needed to inform educational efforts to enhance prevention. Quantifiable outcomes can provide the necessary incentives to drive policy decisions in support of small rural communities like those in East Texas. Evidence-based medicine works well only when data are collected regularly to inform interventions and implementation strategies [34].

This study highlighted perceived challenges and distinctive perspectives from several stakeholder groups, providing a much richer understanding of the larger problem. The study is also subject to a potentially important limitation. Most of the participants were active in the community, addressing SUD needs or advocating for better services; this was especially the case with counselors and those representing CBOs. Thus, we may have missed the voices of stakeholders who are not engaged actively in the struggle to combat SUD. This limitation does not, however, detract from the richness of the data we gathered from various stakeholders seeking to improve care and services for people living with SUD in East Texas.

## 5. Conclusions

This project engaged diverse stakeholder groups, including people living with SUD and their families, to identify community-driven needs and priorities regarding substance use and misuse in East Texas. Based on this input, we describe the social and cultural contexts in which substance use manifests in the region, how the community experiences and copes with the problem, and which needs should be prioritized. Distinct geographic features were identified as contributors to easy, cheap access to drugs in the region. Limited access to behavioral health treatment and recovery services as well as lack of training/educational opportunities related to SUD prevention and treatment pose major challenges in the region. Service coordination and support for families and caregivers must be made more robust. Finally, participants presented clear and distinct differences rooted in their role-specific perceptions of the SUD problem through their professional lenses. The need to develop effective interventions for SUD treatment, interventions to help individuals achieve mental health resiliency, community reentry programs after incarceration, engagement of families/caregivers in SUD prevention and treatment, and training to help law enforcement officers more effectively manage individuals with SUD were prioritized as major needs. Data regarding SUD in East Texas that would inform research and services are scarce. The results of this exploration should help relevant stakeholders prioritize SUD prevention, treatment, and recovery needs and inform programs and policies related to SUD in rural East Texas.

## Figures and Tables

**Table 1:** Distribution of Participants in Focus Group Discussions.

Group	Male	Female	Total
Families	0	7	7
Law Enforcement	3	6	9
People living with SUDs or in recovery	7	3	10
CBO representatives	2	8	10
Counselors/Social Workers	1	5	6
Clinicians	1	3	4
Total	14	32	46

## Data Availability

Not applicable.

## Appendix A

**Table A1:** Illustrative quotes from the major themes on SUD related needs identified by community stakeholders.

Theme	Illustrative Quotes
Access to SUD treatment and recovery resources	It’s really hard to get into a treatment center, of course unless you can pay for it. But I mean people that can’t afford, it’s just, you know, you have to go on a wait list and stuff like that.… What they do is while they’re on the wait list is they go out and do some more of whatever. [Family member]
	They don’t know where to turn to after they get released out of prison. They’re just let back on the street and a lot of them don’t go…have different resources. No assistance, no guidance, no one to assist them in navigating what that looks like. And they end up right back into the same environment, running around with the same people, doing the same things that they were doing trying to maintain. [Law enforcement]
	We have 23 counties and at this point as far as state-funded beds we have one for males and females. We have some for women and children, but the services are limited. And we stay at capacity here. We stay at capacity here. [Counselors]
	It’s baffling to me just how easy it is to find XYX drugs. And the sad thing is about that is that, on the flip side for recovery, from what I’ve seen and what—I’ve asked others around me—it’s hard to find organizations or recovery institutions or something like that in this area to assist with recovery that’s outside of the sober living program. [Person living w/SUD]
	I would say that a lot of people with substance use problems they don’t acknowledge it as a medical problem…so they don’t seek care at a primary care office. I think people are also afraid they’ll be blackballed. Like if you have some sort of substance use disorder you’re never going to get your pain treated or something along those lines. So there’s a lot of things we need to overcome as far as culture in order to access care in the appropriate way and let them know that primary care is actually a way to access care. [Provider]
Mental health and resiliency	I also think it would be interesting if instead of just learning about the dangers they could learn about what are some alternative coping skills because I am always shocked and heartbroken when I do presentations to students and I ask them why do you think your peers are drinking. And I always get at least one student who says that they’re trying to shut off their feelings. [CBO #1]
	But these patients with depression, anxiety, bipolar, what have you, they really need an annual drug screen at minimum to assess. Because if we don’t screen for it, if we don’t see it, it’s difficult to treat it. [Provider]
	And how are these people supposed to get and stay sober if they feel like sometimes, all of the odds are against them? And that’s why me and my other two colleagues—I feel like we fight because we don’t want to be statistics. [Person living w/SUD]
	And it‘s frustrating, because I firmly believe that there are people out there who have surrendered or are wanting to surrender and wanting to get clean and sober, and they can’t because they feel like a lot of things are working against them. [Person living w/SUD]
Education, training, and professional development to facilitate SUD treatments	They need some kind of curriculum that actually tells them what addiction is, and whether it’s the scientific part of it, how many years it shaves off of you, or it makes you look old fast, or your organs, and shutting it down, and your thinking—you only get one mind, shaping those things. But I think it just needs to be called what it is. Addiction is a thief. Nobody likes a thief. [CBO #2]
	Kids need education on if we could get our hands on more data with it. Being able to get them I guess more in-depth data and the education to go with that would be a big priority for the world we’re living in right now. [CBO #1]
	We don’t have the people available to teach and treat, I should say. You know, maybe that’s another area that needs to be looked at, is educate people so that we can deal with the issue that we have. [Family member]
	So I mean our officers…I feel like we are trying to get the best training we can. We’re open and we’d love to have as much training as we can to make sure we’re as prepared as we can be. It’s always a challenge.… I just don’t have the people or the manpower. We do our best to rotate people through there and get as much done as we can. And I think they’re well prepared but there are certainly some challenges in getting everybody trained in substance abuse and de-escalation and everything else that’s throw at us, so it’s a big challenge to get it all done. [Law enforcement]
	I would say my challenge is I don’t have the skillset. Almost period, the end. It was just never part of training. I mean we knew how to detox somebody in the ICU or on the floor. But dealing with somebody in a longitudinal way the way Sonja is was just never part of our training and it seems like it’s not as accessible as it needs to be. [Provider]
Care and service coordination	And any time that I have gone to a doctor about it, they just throw a lot of benzodiazepines at you like Xanax or Clonazepam, things like that, and don’t even fully try to help you with the issue at hand, which is—what you need is counseling and talking about your issues and learning—more education about what is going on with you. They would rather just throw a prescription at you and say, “Here you go. I hope that works.” And so, that became—one of my biggest problems was an addiction to benzos. [Person living w/SUD]
	And for me the next step would look like being able to coordinate better care with the ones that have co-occurring disorders, where they’re not having to wait 30 days for an appointment after being clean and sober for that time. You can start making that transition. Because that gap, that gap in between leaving treatment and getting into a local mental health authority kicks them in the teeth every time. [Counselors]
	I look for as a family medicine provider would be to have clear channels, some kind of clear pathways for people who need referral for treatment. It seems like that’s what I’m always stretching for is where does this person go, where does this person belong or where could we get them the help we need. [Provider]
Community/social support for people living with SUD and their families	There’s not very many support services for the families, to teach them what to do, to teach them not to enable and give them support and let them know they’re not out here by themselves.… And so I think there needs to be more resources for families to go and get the support and education on what to do to help their addict or alcoholic person and their family. Or how to help themselves stay sane, you know? [Family member]
	It was a support group that helped us talk about what was really going on, so that we didn’t have to live in a secret. They say we’re as sick as our secrets, and some of us can get pretty sick in those secrets. And it was a safe place. [Family member]
	After you’ve dished out tens of thousands of dollars the first time to get treatment, you aren’t able to do it a second time…there comes a vicious cycle I see in the people that I’m surrounded with, my support group, that are also dealing with family members with addiction.… It’s a family disease, and I don’t think there’s enough information and education about that. [Family member]
	A lot of them don’t know where to turn to, where to get those resources. I don’t know. I don’t know if it’s an education thing with their probation officer if they don’t know where to get the resources from or parole officer. They don’t know where to go to. Then the other issue is accessibility. Some people don’t have the means to get to the places or if it’s a cost associated with it they don’t have the money to pay for it. So if they don’t have the money to pay for it then the service is not available. Then the navigations of it. How do I fill out the paperwork? I may not have identification going on. I may not have any of the credentials that’s needed in order to attain these resources that’s available to me. [Law enforcement]
	It’s frustrating, because from what my experience was, solely, with the exception of the sober living house that I have found, other than that and the 12-step programs, there was nothing to get me sober. I had no support from anybody, and I had to do this on my own and accept that I was gonna be alone on this until I could turn the tables around. And so, it’s frustrating because there’s supposed to be this movement to assist those with substance abuse disorder. [Person living w/SUD]
